# Simvastatin Re-Couples Dysfunctional Endothelial Nitric Oxide Synthase in Experimental Subarachnoid Hemorrhage

**DOI:** 10.1371/journal.pone.0017062

**Published:** 2011-02-23

**Authors:** Mohammed Sabri, Jinglu Ai, Philip A. Marsden, R. Loch Macdonald

**Affiliations:** 1 Division of Neurosurgery, St. Michael's Hospital, Toronto, Ontario, Canada; 2 Labatt Family Centre of Excellence in Brain Injury and Trauma Research, Keenan Research Centre, Li Ka Shing Knowledge Institute of St. Michael's Hospital, Toronto, Ontario, Canada; 3 Department of Surgery, University of Toronto, Toronto, Ontario, Canada; 4 Renal Division, Keenan Research Centre, Li Ka Shing Knowledge Institute of St. Michael's Hospital, Toronto, Ontario, Canada; 5 Department of Medicine, University of Toronto, Toronto, Ontario, Canada; University of North Dakota, United States of America

## Abstract

Reduced endothelial nitric oxide synthase (eNOS) function has been linked to secondary complications of subarachnoid hemorrhage (SAH). We previously found that there is increased eNOS function after SAH but that it is uncoupled, leading to secondary complications such as vasospasm, microthromboembolism and neuronal apoptosis. Here we test the hypothesis that recoupling eNOS with simvastatin can prevent these complications. SAH was created in mice that were treated with vehicle or simvastatin starting 2 weeks before or 30 minutes after SAH. SAH increased phosphorylated eNOS which was prevented by pre- or post-treatment with simvastatin. Simvastatin pre-treatment also prevented the increase in eNOS monomer formation that was associated with SAH, decreased superoxide anion radical production and increased NO. These changes were associated with decreased vasospasm, microthromboemboli and neuronal injury. The data suggest that simvastatin re-couples eNOS after SAH, leading to decreased secondary complications such as vasospasm, microthromboemboli and neuronal injury.

## Introduction

Subarachnoid hemorrhage (SAH) is associated with serious secondary complications such as cerebral vasospasm, delayed cerebral ischemia, cortical spreading ischemia and microthromboembolism [1], [2]. Theories for the development of cerebral vasospasm and these associated secondary complications include either the depletion or excess production of nitric oxide (NO). NO is produced enzymatically by three nitric oxide synthase (NOS) isoforms, namely endothelial, neuronal and inducible NOS or in a non-enzymatic fashion via nitrate-nitrite redox reaction [3]. Under physiological conditions endothelium-derived NO inhibits thrombosis and platelet adhesion and aggregation, endothelial cell apoptosis, smooth muscle hypertrophy and leucocyte adhesion to the endothelium [4]. Physiologically, eNOS is a homodimer that facilitates NO production via electrochemical regulation of electron flow into the ferrous-dioxygen complex. However, under pathological conditions where blood vessels are exposed to oxidative stress or depleted of cofactors such as tetrahydrobiopterin and L-arginine required for normal eNOS function, the ferrous-dioxygen complex uncouples and electron flow may generate superoxide anion radical (O_2_Γ) instead of NO [4]. It has been demonstrated that the increased O_2_Γ can react with endogenous NO to form peroxynitrite (ONOO^−^), which exacerbates oxidative stress and facilitates eNOS uncoupling [5].

We recently demonstrated in mice that injection of blood into the subarachnoid space induced uncoupling of eNOS and that this was associated with secondary complications such as neuronal apoptosis, microthromboembolism, cerebral vasospasm, oxidative stress and reduced NO bioavailability [6]. We hypothesize that this novel mechanism of eNOS uncoupling is a key mediator of the secondary pathological changes of SAH. This study aims to obtain more direct evidence for this mechanism and to determine effects of alleviating it with a 3-hydroxy-3-methylglutaryl coenzyme A reductase inhibitor, simvastatin, which has been demonstrated to improve endothelial function and reduce oxidative stress [7]. If this hypothesis is correct, simvastatin, by eliminating oxidative stress, would re-couple eNOS, restore NO availability and alleviate any secondary complications of SAH that are a result of this mechanism.

## Materials and Methods

### Animals

Experimental protocols were approved by the Institutional Animal Care Committee. eNOS-lacZ mice were generated by utilizing a murine eNOS promoter with the eNOS ATG site mutated to prevent translation [8]. The promoter was then coupled to a lacZ open reading frame. Mice were genotyped using Southern blot analysis of genomic tail DNA. We used 60 endothelial NOS-lacZ transgenic mice of both sexes weighing 19–25 g (2 to 6 months old). Thirty mice (used for either histological studies or molecular biology and biochemistry studies) were randomly assigned to pre-SAH (15 mice) or post-SAH treatment (15 mice) groups. Within each of the treatment groups, 7 to 8 mice were assigned to be treated with simvastatin or vehicle.

### SAH model and simvastatin treatment scheme

SAH was created as previously described with modifications [9]. To eliminate the inconsistency of time used to take blood from tail and prevent coagulation of blood before injection, we induced SAH by pre-chiasmatic cistern injection of non-heparinized blood taken from hearts of donors with the same genetic background as the transgenic mice (C57BL). For the pre-treatment group (n = 15), the animals were injected with simvastatin, 20 mg/kg subcutaneously (dissolved in DMSO, then diluted with 0.9% NaCl with a final concentration of DMSO of 10%), daily for 14 days before the onset of SAH.

For the post-SAH simvastatin treatment group (n = 15, the same dose of simvastatin was injected 30 minutes after SAH creation, and then daily for two days. For the vehicle group, treatment with equivalent volumes of vehicle was administered daily for either 14 days or 2 days. Allocation to groups was sequential with a treated and a vehicle animal operated each day. In some experiments, we also included naive animals (C57BL) as untreated controls, which were anaesthetized and euthanized as in other groups. Mice were sacrificed 48 hours after surgery. They were perfused through the left cardiac ventricle with NaCl, 0.9%, 10 ml, followed by 150 ml 4% paraformaldehyde in phosphate-buffered saline (PBS). Brains were removed and fixed in 4% paraformaldehyde for 48 hours. Brain blocks were embedded in paraffin. Sections 7 µm thick were cut using a microtome (Leica, Wetzlar, Germany) for histological studies.

### Hematoxylin and eosin staining and vasospasm measurement

The staining procedure was as described [6], [9]. Paraffinized sections were deparaffinized with xylene and rehydrated through a decreasing gradient of ethanol solutions. Slides were stained with hematoxylin and eosin, coverslipped with xylene-based mounting medium (Permount, Sigma) and viewed under a light microscope. Slides were scanned with a digital scanner (MIRAX scanner, Carl Zeiss, Göttingen, Germany) and viewed with MIRAX software (Zeiss). The lumen area and thickness of the middle cerebral artery (MCA) was quantified by a blinded observer at 200x magnification using Image J (National Institutes of Health [NIH], Bethesda, MD). The sizes of images were calibrated based on their magnification and MCA lumen was outlined using the free hand tool to obtain lumen area. Artery wall thickness was measured at 4 equally spaced points along the artery circumference and averaged to obtain artery thickness.

### LacZ staining and detection

Brain sections were excised and dissected into 3 mm sections to maximize surface area [6], [8]. Sections were rinsed in PBS and then immersed in fixative solution (0.2% glutaraldehyde, 2% formaldehyde, 5 mmol/L EGTA, 2 mmol/L MgCl_2_, and 100 mmol/L Na_2_H_2_PO_4_, pH 7.3). Sections were again rinsed in PBS, blotted, and immersed in aqueous X-Gal solution (1 mg/ml 5-bromo-4chloro-3-indolyl-B-D-galactopyranoside, pH 7.3, Boehringer Mannheim, Laval, Canada) for 16 hours in the dark at room temperature. Tissue was embedded in paraffin, sectioned (7 µm) and counterstained with neutral red (Sigma-Aldrich, St. Louis, MO, USA) [8]. Localization and expression of β-galactosidase was assessed by light microscopy by two independent observers blinded to the type and group of the mice.

### Peroxynitrite assay and anti-nitrotyrosine

Tissue sections were deparaffinized with xylene and dehydrated in graded ethanol solutions. Antigen was retrieved by heating the sections for 25 minutes in 0.01 mmol/L sodium citrate (pH 6.0) at 96°C [6]. Endogenous peroxidase activity was quenched by incubating the sections for 30 minutes in 0.3% H_2_O_2_ in water. Sections were blocked with 10% normal goat serum in PBS for 20 minutes to attenuate non-specific IgG binding. Polyclonal rabbit anti-mouse nitrotyrosine antibody (rabbit polyclonal 1∶200) was used as a marker for peroxynitrite (ONOO^−^) and was added to the sections and incubated for 60 minutes. A secondary biotinylated antibody (goat anti-rabbit) was added and specimens for 30 minutes. The VIP reaction was then performed utilizing the VECTASTAIN ABC Kit.

### NO, superoxide anion radical detection

Superoxide anion radical (O_2_^−^) and NO were detected in homogenized fresh or deep frozen brain tissue using spectrophotometric methods [6]. The cell-permeable fluorophore 4,5-diaminofluorescein-2-diacetate (DAF-2DA, Alexis Biochemicals, Gruenberg, Germany) was used to detect NO and a chemiluminescence probe, 2-methyl-6-(*p*-methoxyphenyl)-3,7-dihydroimidazo[1,2-]-pyrazin-3-one (MCLA) for O_2_^−^ detection. Homogenized brain tissues were incubated with either 10 µmol/L DAF-2DA for 30 minutes or 4 µmol/L MCLA in transparent 96-well plates for 10 minutes (Fisher, Ottawa, ON, Canada) at room temperature in the dark. DAF-2DA was excited at 495 nm and emission read at 515 nm in a spectrofluorometer (SpectraMAX-Gemini, Molecular devices, Sunnyvale, USA). The luminescence signal of MCLA was read directly at 495 nm. All experiments were repeated 3 times. To test the specificity of MCLA, increasing concentrations of superoxide dismutase (SOD) (1–10 U/ml) was used to reveal a concentration dependent MCLA luminescence signal (data not shown). Since MCLA crosses cell membranes, O_2_^−^ was detected from both intra- and extracellular sources.

### Western blots for eNOS and iNOS

Brain tissue (n = 7–8 per group) was acutely excised and stored at −80°C. Tissue was homogenized in 300 µL 1% RIPA buffer with 0.1% protease inhibitor, and centrifuged at 13,000 rpm for 12 minutes at 4°C. Protein was quantified using the Bradford method, where RIPA buffer was used as the blank standard. 30 µg protein was loaded and separated by electrophoresis on 8% sodium dodecyl sulphate - polyacrylamide gels (SDS-PAGE) and transferred onto nitrocellulose membrane. We used Ponceau S and Gel Code to stain the membrane and gel, respectively. Blots were incubated with 5% milk for 60 minutes, followed by incubation with primary monoclonal antibodies (1∶1000 dilution) against phosphorylated S1177-eNOS (BD Biosciences, San Jose, CA, USA), eNOS (Cell signaling Danvers, MA, USA) and iNOS (Abcam Inc, Cambridge, MA, USA). After washing in PBS, membranes were incubated in horse radish peroxidase conjugated goat anti-mouse antibody (Abcam) at a dilution of 1∶1000 for 50 minutes at room temperature. Reactions were developed with ECL reagent mix (Amersham Biosciences, UK). Protein intensities were quantified by densitometric analysis utilizing Image J software (NIH).

For eNOS monomer and dimer detection, we used low-temperature SDS-PAGE [10], [11]. Samples were subjected to SDS-PAGE on 8% gels that were kept in an ice bath at 4°C. The gels were then blotted into nitrocellulose membranes and blocked. The membranes were incubated with primary antibodies against eNOS (Cell Signalling Danvers, MA, USA) and the remainder of the procedure was identical to the standard Western blot steps.

### Immunohistological staining for fibrinogen, caspase-3 and NeuN

After deparaffinization and rehydration, antigen was retrieved by heating the sections for 25 minutes in 0.01 mmol/L sodium citrate (pH 6.0) at 96°C. For fibrinogen staining, endogenous peroxidase activity was quenched by incubating the sections for 30 minutes in 0.3% H_2_O_2_ in water. Sections were blocked with 10% normal goat blocking serum in PBS for 20 minutes and incubated with fibrinogen first antibody (chicken anti-rat 1∶200, Immunology Consultants Laboratory, Newberg, OR) for 60 minutes. They were washed with PBS and incubated with secondary biotinylated anti-body (goat anti-chicken, Millipore) for 30 minutes. Staining was visualized with VIP using the VECTASTAIN 7 ABC Kit (Vector) and counterstained with 0.5% methyl green.

For caspase-3 and NeuN staining, after antigen retrieval, sections were permeabilized with 0.3% Triton X-100 for 15 minutes and then incubated with 10% normal goat serum, 1% bovine serum albumin and 0.1% sodium azide for 60 minutes. Primary antibodies were monoclonal anti-NeuN (1∶400, Invitrogen, Carlsbad, CA, USA) and rabbit anti-human active cleaved caspase-3 (1∶400, BD Pharmingen, Franklin Lakes, NJ, USA). Secondary antibodies were Alexa Fluor 568 conjugated goat anti-mouse for NeuN (1∶1000, Invitrogen) and Alexa Fluor 488 conjugated goat anti-rabbit for caspase-3 (1∶1000, Invitrogen). After incubation, sections were cover slipped with anti-fading mounting medium and sealed with nail polish. Slides were viewed with a confocal microscope and images taken using constant parameters.

### TUNEL and Fluoro-Jade staining

Apoptosis was assessed using terminal deoxynucleotidyl transferase dUTP nick end labeling (TUNEL, DeadEnd Flurometric kit, Promega, WI, USA). Fluoro-jade B (Histo-Chem Inc, Jefferson, AR, USA) staining was performed according to a previously published protocol [9]. In brief, after deparaffinization and subsequent rehydration, the slides were incubated in 0.06% potassium permanganate (VWR International, Strasbourg, France) for 15 minutes. Slides were then rinsed in deionized water and immersed in 0.001% fluoro-jade B working solution (0.1% acetic acid) for 30 minutes. Then they were washed and dried in an incubator (60°C) for 15 minutes. Sections were cleared in xylene and coverslipped with a non-aqueous, low fluorescence, styrene based mounting medium (DPX, Sigma). Slides were viewed under a confocal microscope and images taken using constant parameters (laser power, exposure time and pinhole size).

### Statistical analysis and data quantification

All data are presented as means ± standard deviation (SD). Data were compared within groups over time and between groups at each time by analysis of variance (ANOVA) or Student's t-test for continuous variables. *P*<0.05 was considered significant.

To keep the quantification on staining consistent, we pre-determined five fixed symmetrical areas for cerebral cortex (three for hippocampus) on a proper coronal section chart of the mouse brain (Paxinos and Franklin, 2001). For fibrinogen staining (200x magnification), we took one image from each fixed area (10 images from cerebral cortex, 6 images from hippocampus) and counted all microthromboemboli observed in the section. To determine positive staining in TUNEL and casepase-3/NeuN stained cerebral cortex, we used higher magnification (400x), and took 3 images from each of the fixed areas (30 images for both sides of cerebral cortex). For the hippocampus, we counted all the positive cells for TUNEL and caspase-3 staining, along all regions of the hippocampus (dentate gyrus, CA3 and CA1) under microscope. All counting was done by blinded researchers.

## Results

### Simvastatin down-regulates phosphorylated eNOS

X-gal staining was performed to localize the activity of LacZ/eNOS in brains and cerebral arteries from these mice. We previously found that X-gal staining was localized to the endothelium of large arteries. SAH substantially increased the expression of eNOS as compared to saline-injected controls [6]. In the current study, we therefore compared end points between simvastatin- and vehicle-treated mice with SAH.

**Figure 1:**
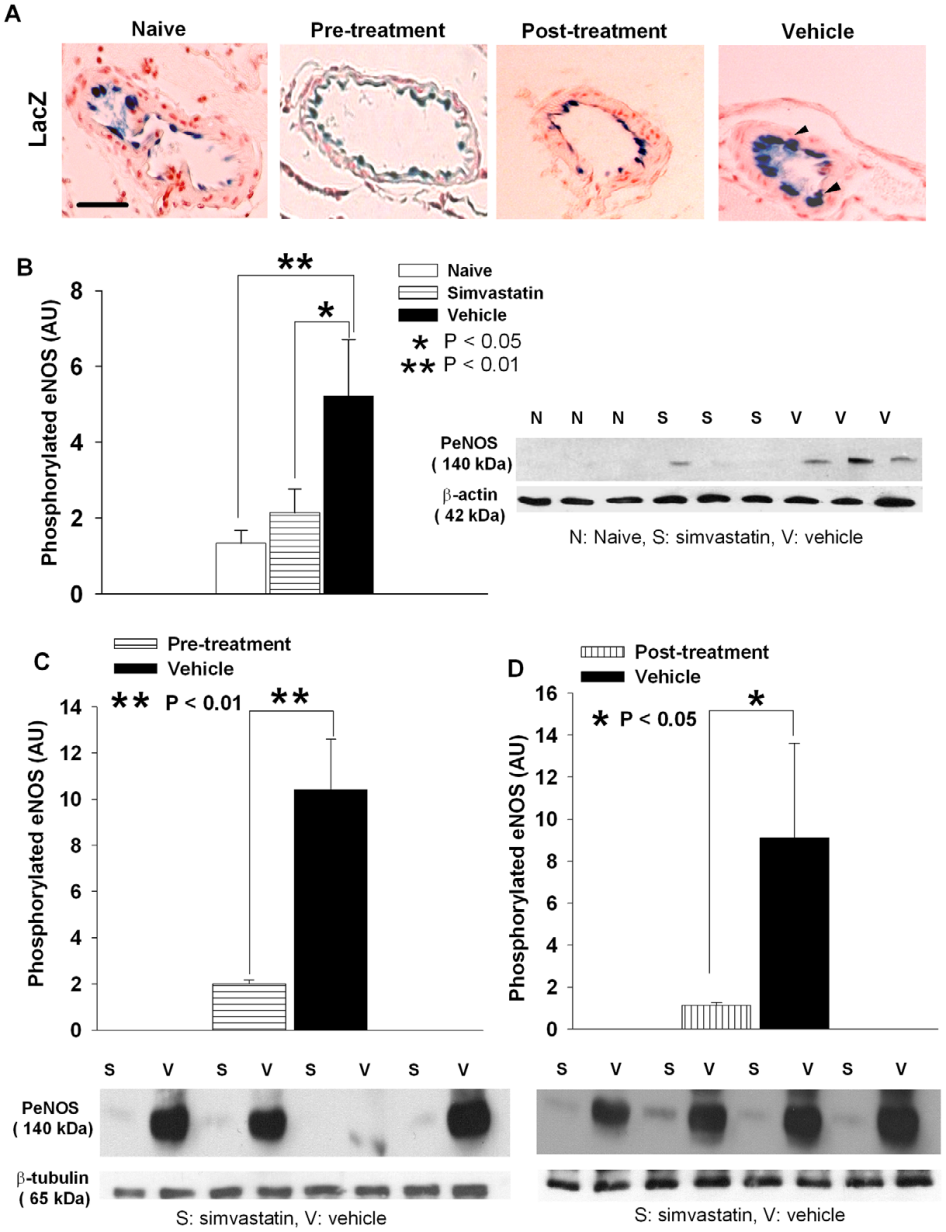
Simvastatin decreases eNOS expression. **A.** Representative images of LacZ staining of the middle cerebral artery (MCA) from naïve, vehicle, pre- and post-SAH simvastatin-treated animals. A thick densely stained LacZ positivity appears in the endothelial layer of the MCA from vehicle animals (arrow heads). This is significantly reduced in both pre- or post-SAH treatment groups, to a similar level in naïve group. Scale bar is 50 µm. **B.** Quantification of phosphorylated eNOS from naïve, vehicle or simvastatin pre-SAH treated groups. Simvastatin treatment significantly reduced phosphorylated eNOS almost to the same level in the naïve animals. A representative blot is shown for each group. **C** and **D**. Quantification of phosphorylated eNOS from pre-SAH or post-SAH treated groups, respectively. Both pre- and post-SAH treatment significantly reduced phosphorylated eNOS expression (*P*<0.01 for pre-treatment, *P*<0.05 for post-treatment). A representative blot is shown for each treatment group under corresponding bar graphs. All data are mean ± SD.

In vehicle-treated animals, there was thick, dense LacZ staining in the endothelial layer of the MCA (Fig. 1A). This was significantly reduced by pre- or post-treatment with simvastatin (Fig. 1A). We investigated this finding further using Western blotting and found significant down-regulation of the expression of the phosphorylated form of eNOS in both simvastatin-treated groups (Figs. 1B, 1C and 1D). In the initial batch of experiments, the expression of phosphorylated eNOS in naïve mice was negligible (Fig. 1B); therefore we eliminated this group in the subsequent experiments shown in Fig. 1C and 1D. Densitometry quantification of phosphorylated eNOS was 2.0±0.2 and 10.4±2.2 for pre-SAH simvastatin-treated and vehicle-treated animals, respectively (Fig. 1C, *P*<0.01). Phosphorylated eNOS level was 1.1±0.1 for post-SAH simvastatin-treated and 9.1±4.5 for vehicle-treated animals (Fig. 1D, *P*<0.05).

### Simvastatin increases eNOS dimer/monomer ratio and NO production

**Figure 2:**
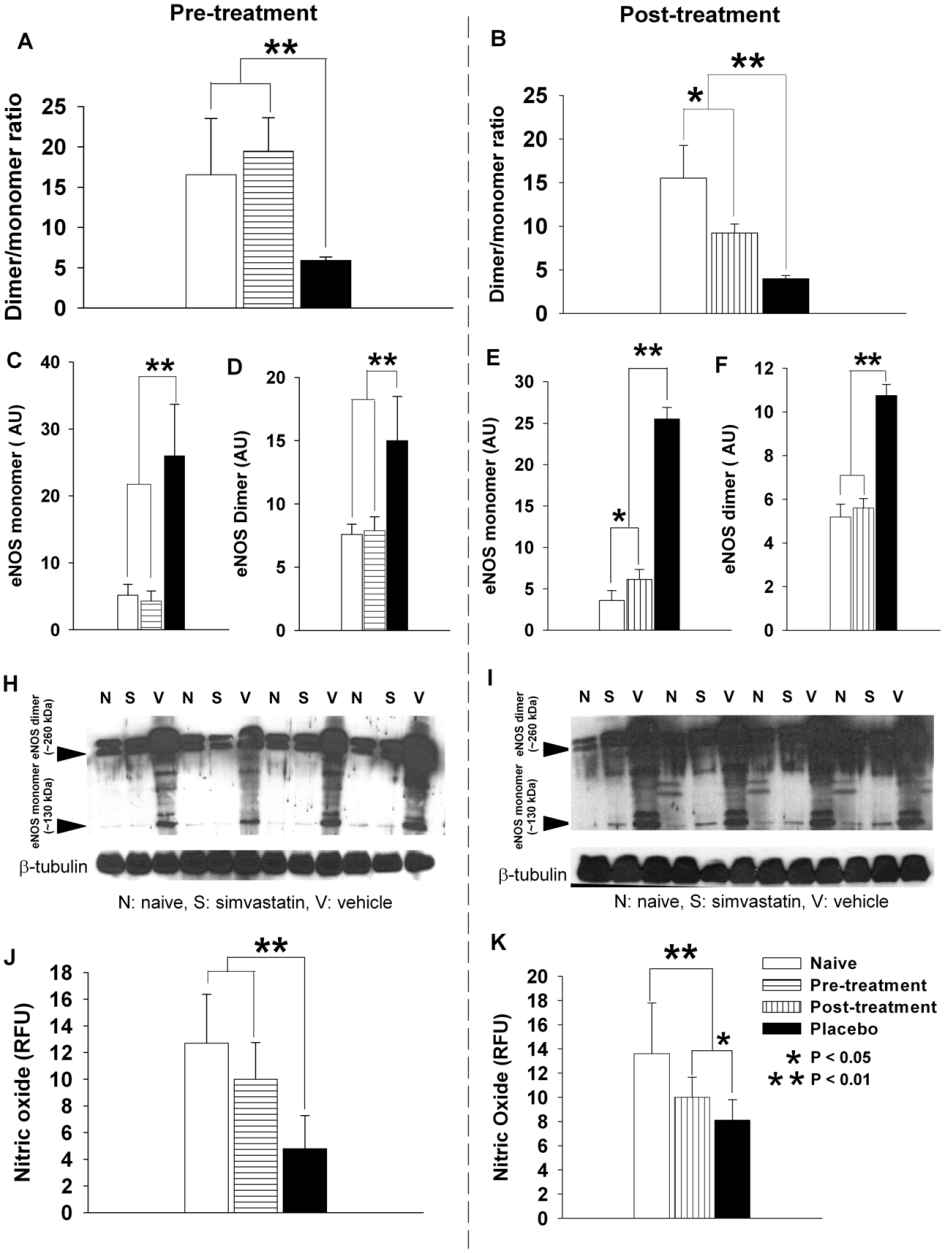
Simvastatin increases eNOS dimer/monomer ratio and NO production. **A** and **B**. Dimer/monomer ratio of eNOS protein expression for pre- and post-SAH treated groups, respectively. Pre-SAH treatment with simvastatin significantly increases dimer/monomer ratio to the naïve level (*P>*0.05). Post-SAH treatment also markedly increases the ratio, but to a lesser extent (*P*<0.05 as compared to naïve controls). Vehicle treated animals exhibit significantly lower dimer/monomer ratio than both naïve or the two simvastatin treated groups (*P*<0.01). **C** to **F**. Western blot quantification of separate monomer and dimer protein expression in pre- (**C** and **D**) and post-SAH (**E** and **F**) simvastatin treated animals. In pre-treated animals, both eNOS monomer and dimer expression were decreased by simvastatin to the same level as in naïve animals. They were significantly lower than that in vehicle treated animals (**C** and **D**, *P*<0.01 for both monomer and dimer). Similarly, in post-treated animals, both eNOS monomer and dimer expression were decreased by simvastatin as compared to that in vehicle treated animals (*P*<0.01 for both). However, while eNOS dimer was reduced by post-SAH treatment of simvastatin to the same level as in naïve animals (**F**), the eNOS monomer was still significantly higher than that in naïve controls (**E**, *P*<0.05). Representative blots of monomer and dimer protein expression are shown in **H** (for pre-treatment) and **I** (for post-treatment). Spectrometric measurement of NO shows pre-SAH treatment with simvastatin restored NO production to the level seen in naïve controls, which is significantly higher than vehicle treated (**J,** *P*<0.01). In contrast, post-SAH simvastatin treated animals shows a significantly increased NO production as compared to vehicle treated (**K**, *P*<0.05), but not to the same level as in naïve animals (**K**, *P*<0.01). All data are mean ± SD. Legend in **K** is for all the charts in the figure.

We previously indirectly showed that SAH increased eNOS expression and O_2_Γ concentration in the brain, but decreased NO availability, suggesting uncoupling of eNOS [6]. We now used a direct measurement of eNOS uncoupling, namely, eNOS dimer/monomer ratio [10], [11]. SAH was associated with a significant increase in both eNOS dimer and monomer as compared to naïve controls (Fig. 2C to 2F, *P*< 0.01). However, the relative proportion of dimer, thus potentially functional eNOS, was significantly lower in SAH than in naïve controls (eNOS dimer/monomer ratio was 5.9±0.4 and 4.0±0.3 for SAH, 16.5±6.9 and 15.5±3.8 for naive controls in pre-treatment and post-treatment experiments, respectively, Fig. 2A and 2B, *P*<0.01). Treatment with simvastatin starting before SAH completely reversed the decreased ratio of dimer/monomer to naïve level (*P>*0.05 compared to naïve group, Fig 2A). In comparison, post-SAH treatment with simvastatin also increased the dimer/monomer ratio as compared to vehicle treatment but not to the level of naïve controls (*P*<0.05 compared to naïve, Fig. 2B). This was directly related to the significantly higher than normal level of monomer expression seen in the post-SAH simvastatin-treated animals (*P*<0.05, Fig. 2E). Nevertheless, all naive and simvastatin-treated animals had a significantly higher dimer/monomer ratio than vehicle-treated SAH (*P*<0.01, Fig. 2A and 2B).

The reversal towards normal of the eNOS dimer/monomer ratio by simvastatin treatment correlated with increased NO (Fig. 2J and 2K). Pre-SAH treatment completely restored NO production to that in naïve level (Fig. 2J, *P* *>*0.05 compared to naïve controls, but *P*<0.01 compared to vehicle treated). Post-SAH treatment with simvastatin partially restored NO production (Fig. 2K, *P*<0.01 compared to naïve controls, but also *P*<0.05 compared to vehicle treated). Simvastatin not only prevented the SAH-induced increase in eNOS dimer to naïve level (Fig 2D and 2F), but also effectively reduced eNOS monomer to (pre-treatment Fig 2C) or near (post-treatment Fig 2E) the naïve level. These data support the hypothesis that simvastatin re-couples eNOS to a normal functional state.

### Simvastatin reduces O2Γ, ONOOΓ and iNOS

**Figure 3:**
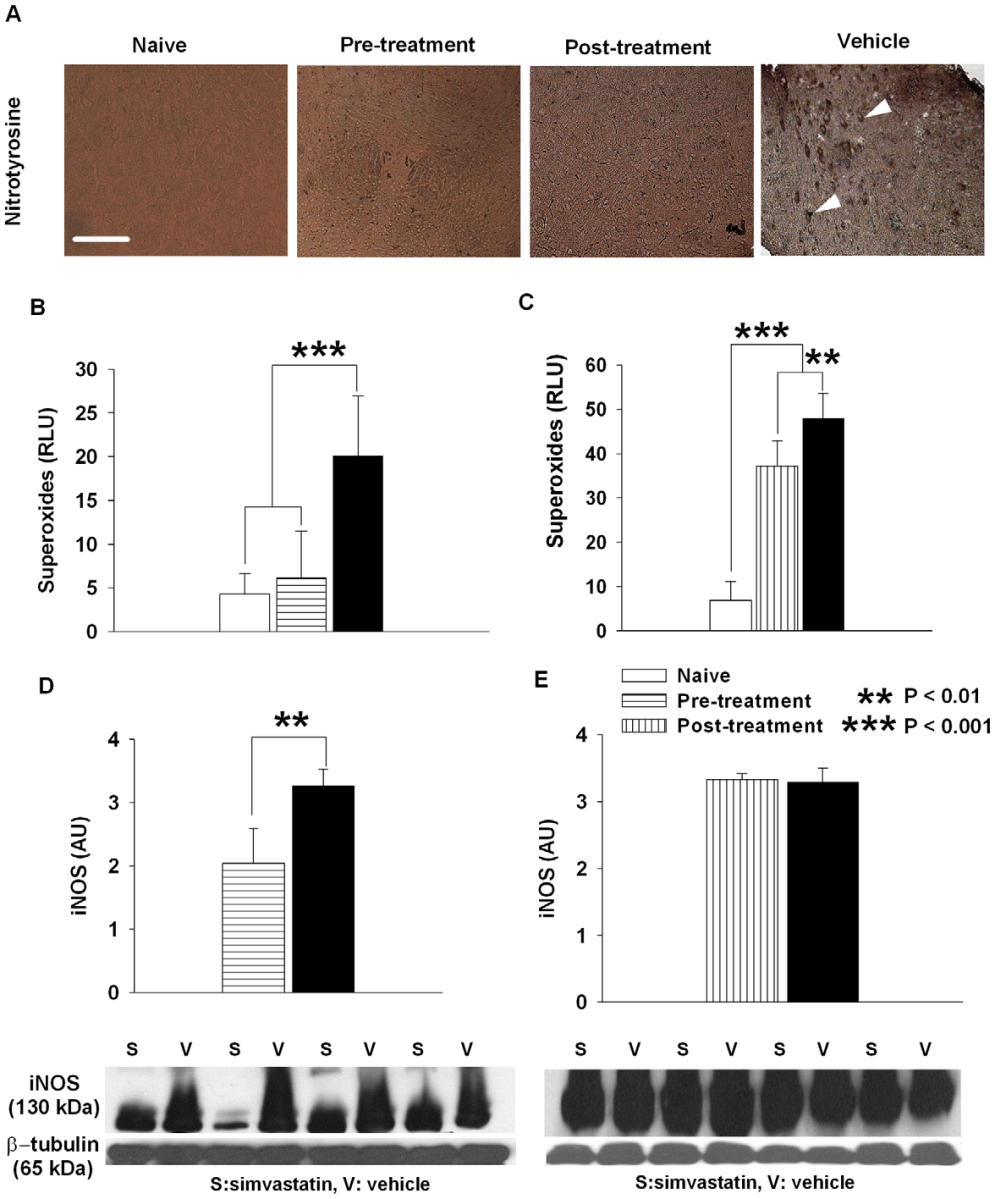
Simvastatin decreases peroxynitrite, superoxide and iNOS production. **A**. Representative images of nitrotyrosine staining. In vehicle treated animals, patches of densely stained nitrotyrosine appear in the brain parenchyma (arrow heads). In comparison, both simvastatin treated groups show a substantial reduction in nitrotyrosine deposits. There is no nitrotyrosine deposit in the naïve group. Scale bar is 150 µm. **B** and **C**. Spectrophotometric measurement of O_2_^−^ shows markedly reduced production of O_2_^−^ from pre-SAH (*P*<0.001) and post-SAH simvastatin treated groups (*P*<0.01) as compared to vehicle treatment. However, only pre-SAH but not post-SAH simvastatin treatment reduce production of O_2_^−^ to the naïve level (*P>*0.05 for pre-SAH and *P*<0.001 for post-SAH compared to naïve controls). **D** and **E.** Western blot quantification of iNOS protein expression show that iNOS protein was significantly decreased by pre- (**D**, *P*<0.01) but not post-SAH (**E**, *P>*0.05) simvastatin treatment as compared to vehicle treated animals. A representative blot is shown for each treatment group under corresponding bar graphs. All data are mean ± SD. Legend in **E** is for all the charts in the figure.

SAH induces perivascular oxidative reactions probably mainly due to oxidation of extravascular hemoglobin [12], [13]. We found that SAH increased O_2_Γ and ONOOΓ production and iNOS expression, all of which may contribute to uncoupling of eNOS [6]. Statins were shown to improve endothelial function and reduce oxidative stress [7]. Here we found that simvastatin treatment either given pre- or post-SAH markedly reduced O_2_Γ (Fig. 3B for pre-SAH, *P*<0.001; 3C for post-SAH treatment, *P*<0.01) and ONOOΓ production (Fig. 3A) as compared to vehicle treatment. However, only pre-treatment of SAH with simvastatin reduced O_2_Γ production to the naïve level (*P*>0.05 Fig. 3B). O_2_Γ production is still very high in post-SAH treated mice compared to naïve (*P*<0.001 Fig. 3C). Similarly, only pre-treatment of SAH with simvastatin reduced iNOS expression (Fig. 3D for pre-SAH, *P*<0.01, 3E for post-SAH treatment, *P*>0.05) as compared to vehicle.

These results show that SAH causes uncoupling of eNOS, which in turn decreases NO availability, but increases O_2_Γ and ONOOΓ concentrations in the brain. This mechanism may contribute to secondary pathology associated with SAH such as vasospasm, microthromboembolism and neuronal death [9]. We tested this hypothesis by determining if simvastatin treatment alleviates vasospasm, microthromboemboli and neuronal cell death [1], [2].

### Simvastatin alleviates vasospasm and microthromboembolism

**Figure 4:**
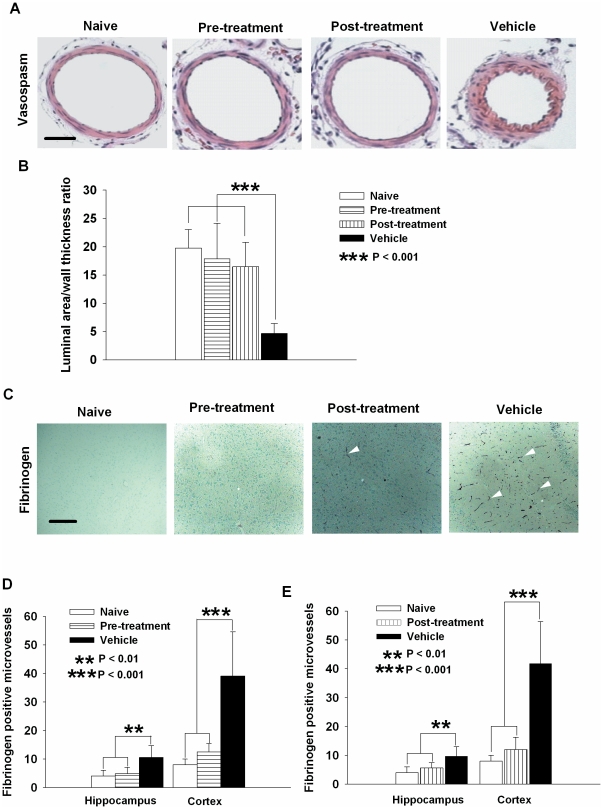
Simvastatin decreases cerebral vasospasm of the middle cerebral artery and reduces microthromboemboli after SAH. **A.** Representative images showing vasospastic MCA in vehicle treated mice, as compared to larger diameter MCAs from the two simvastatin treated groups that are similar to MCA in naïve group. Scale bar is 50 µm. **B**. Both pre- and post-SAH simvastatin treatment significantly increases the lumen area/wall thickness ratio of MCA as compared to vehicle treatment (*P*<0.001). The ratios in both treatment groups are not different from that in naïve control group (*P*>0.05). A small lumen area/wall thickness ratio indicates vasospasm. **C**. Representative images from immunohistochemical staining of fibrinogen show the presence of numerous microthromboembli in SAH animals treated with vehicle (arrow heads) but only few in animals treated with simvastatin either pre- or post-SAH, and none in the naïve controls. Scale bar is 100 µm. **D** and **E**. Both pre-SAH and post-SAH treatment with simvastatin significantly decrease microthromboemboli in both cerebral cortex (*P*<0.001) and hippocampus (*P*<0.01) as compared to vehicle treated group. There was no significant difference between pre-SAH or post-SAH simvastatin treated groups as compared to naïve controls in either brain regions quantified (*P>*0.05). All data are mean ± SD.

Vasospasm of the anterior and MCA and microthromboemboli throughout the brain are observed in the mouse model of SAH [6], [9]. In keeping with prior studies, we found that vehicle-treated SAH animals had vasospasm of the MCA with a lumen/wall thickness ratio of 4.7±1.7 (Fig. 4B). Both pre- and post-SAH treatment with simvastatin alleviated vasospasm (lumen/wall thickness ratio 17.8±6.3 for pre-treatment, 16.5±4.3 for post-treatment, Fig. 4B, *P*<0.001 compared to vehicle). These are not significantly different from that in naïve controls (19.8±3.3, *P>*0.05 Fig. 4B). Microthromboemboli were detected by fibrinogen staining and scattered throughout the brain in vehicle-treated SAH animals (Fig. 4C to 4E). Both schemes of simvastatin treatment eliminated most microthromboemboli in cerebral cortex and hippocampal regions to naïve level (Fig. 4D for pre-treatment, 4E for post-treatment, *P*<0.01∼0.001 compared to vehicle).

### Simvastatin prevents neuronal death

**Figure 5:**
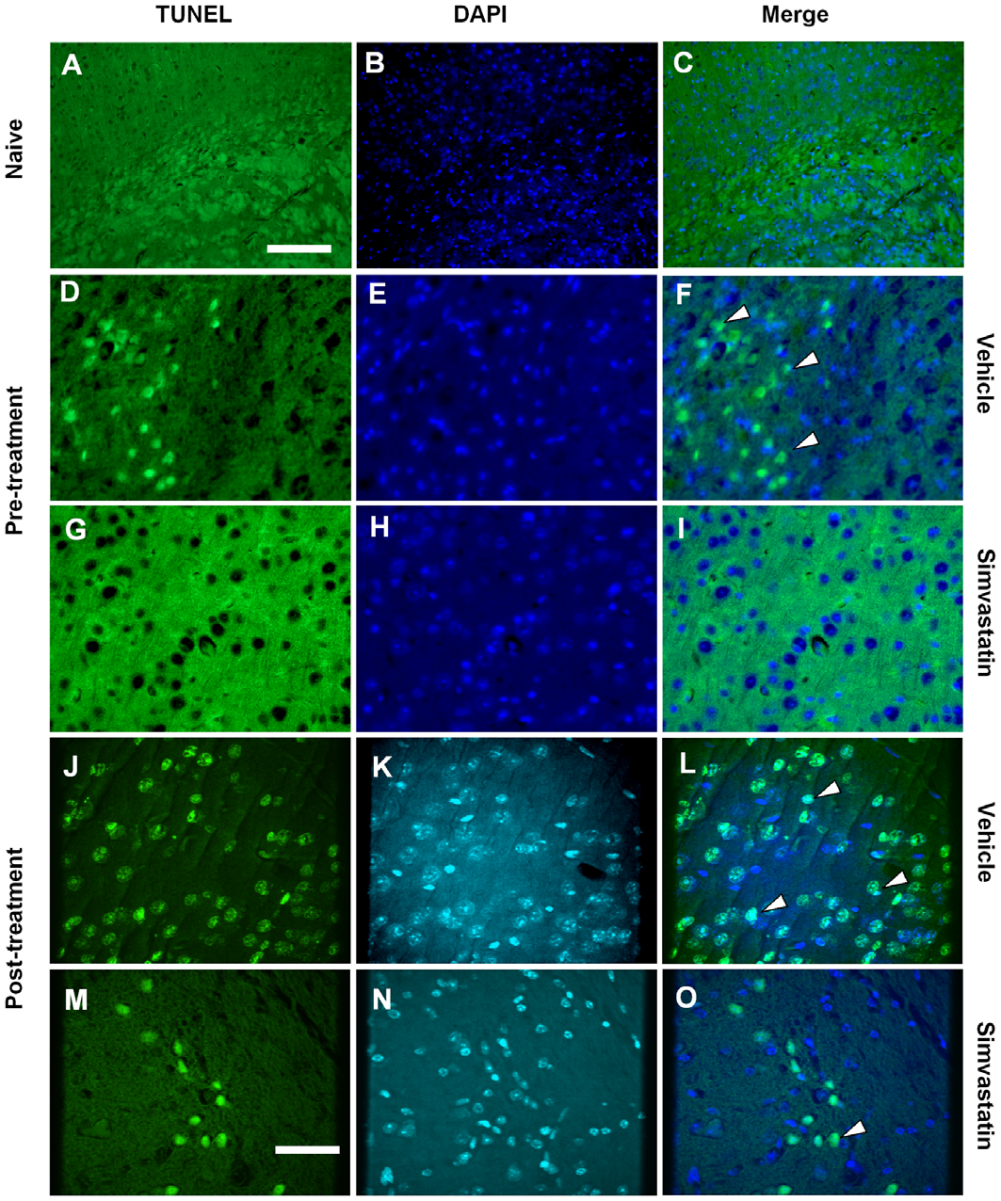
Simvastatin reduces TUNEL positive cells after SAH. Representative images shown are from cerebral cortex. Vehicle-treated animals show numerous TUNEL positive cells (**F** and **L**, arrow heads). In comparison, there are fewer positive cells in post-SAH simvastatin treated animals (**O**, arrow heads), and almost no positive TUNEL positive cells in the pre-treatment mice (**I**), none in naïve mice (**C**). Scale bars are 200 µm in **A** to **C**, 50 µm in **D** to **O**.

**Figure 6:**
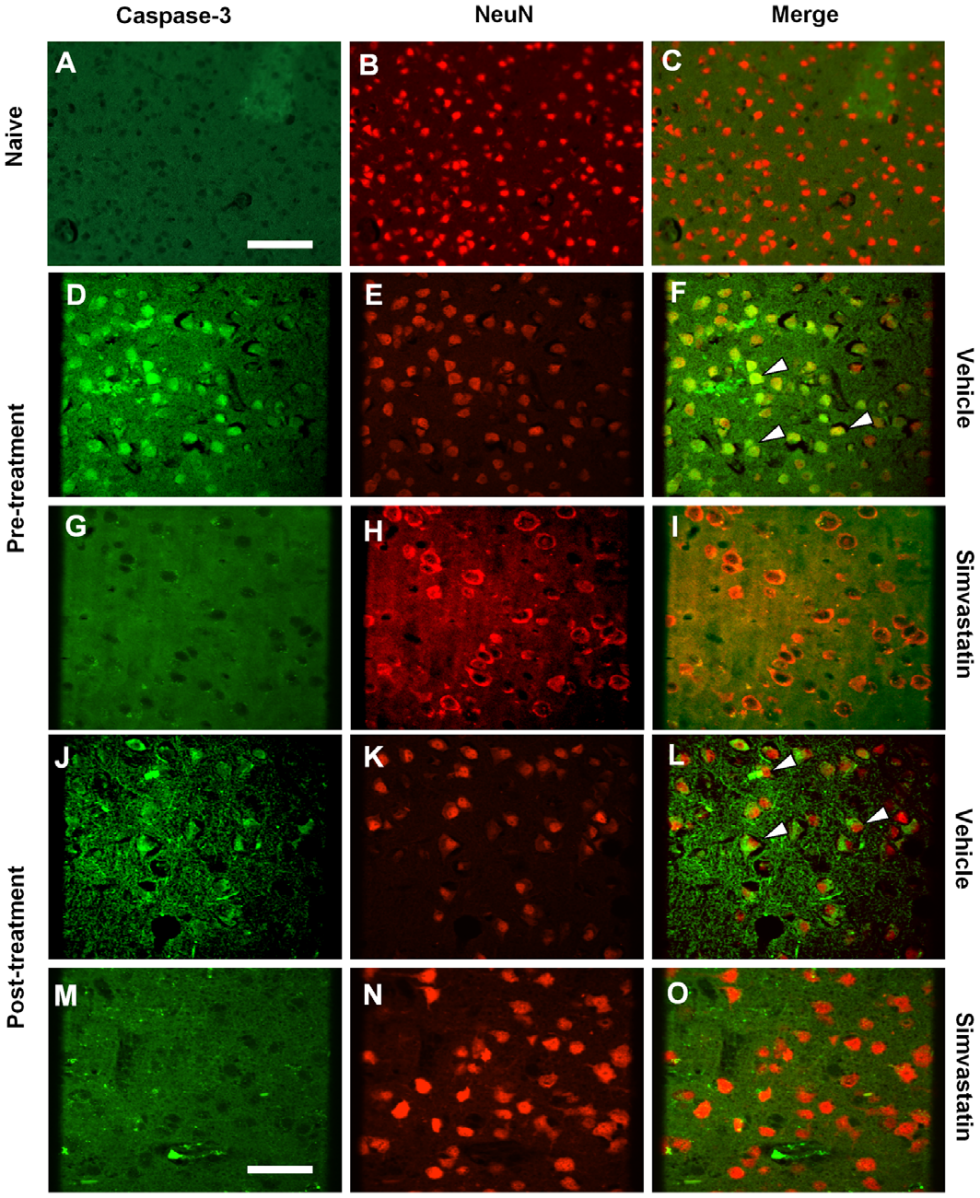
Simvastatin reduces caspase-3/NeuN positive cells after SAH. Representative images shown are from cerebral cortex. Vehicle treated animals show numerous caspase-3/NeuN positive cells (**F** and **L**, arrow heads). In comparison, there are fewer positive cells in post-SAH simvastatin treated animals (**O**), and almost no positive caspase-3/NeuN positive cells in the pre-treatment mice (**I**), none in naïve mice (**C**). Scale bars are 100 µm in **A** to **C**, 50 µm in **D** to **O**.

**Figure 7:**
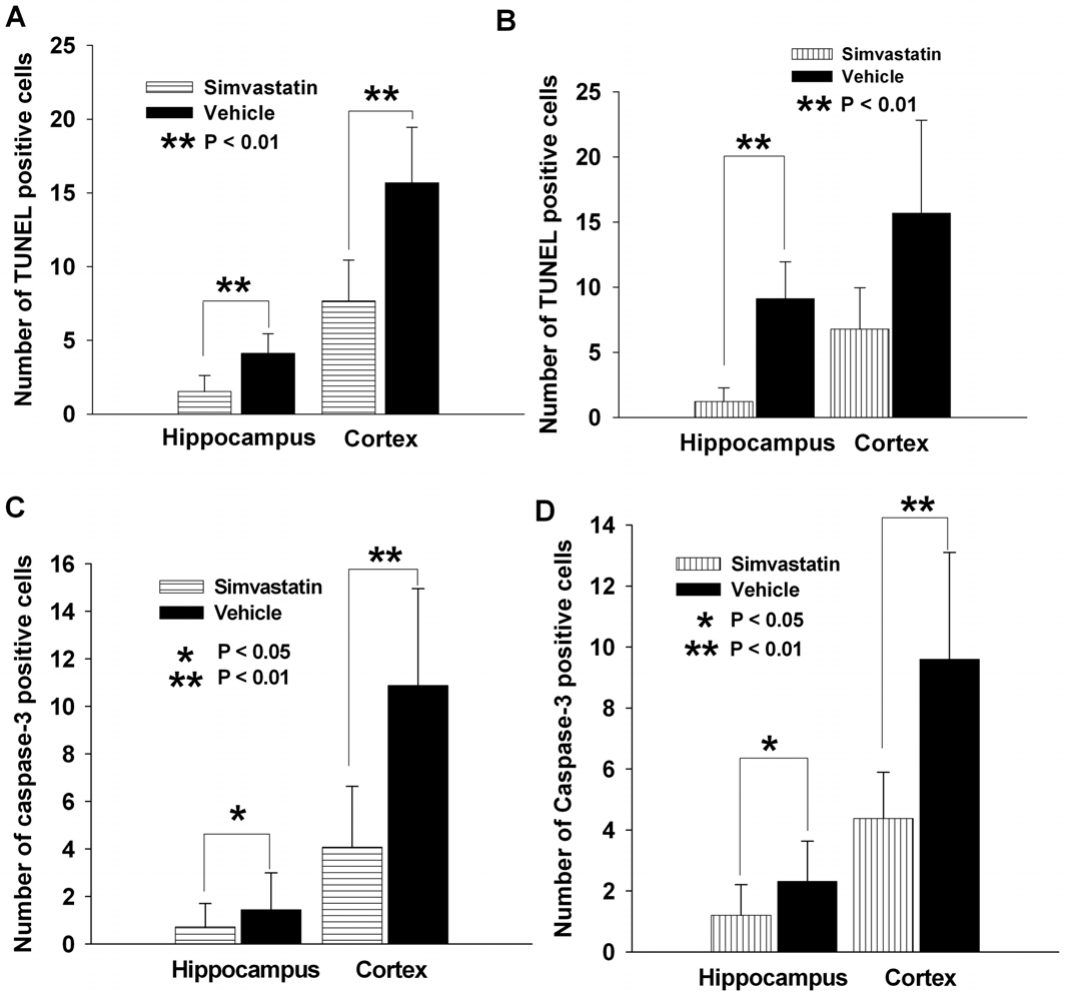
Quantification of TUNEL and caspase-3/NeuN positive cells in cerebral cortex and hippocampus. Pre-treatment of simvastatin significantly reduces the number of both TUNEL (**A**, *P*<0.01 for both cortex and hippocampus regions) and caspase-3/NeuN positive cells (**C**, *P*<0.05 in hippocampus, *P*<0.01 in cortex) as compared to vehicle treatment. Similarly, post-treatment of simvastatin significantly reduces the number of both TUNEL (**B**) and caspase-3/NeuN positive cells (**D**) in brain area of hippocampus (*P*<0.01 for TUNEL, *P*<0.05 for caspase-3/NeuN staining). However, in cerebral cortex, post-treatment with simvastatin only markedly reduced the number of caspase-3/NeuN positive cells (**D**, *P*<0.01) but not TUNEL positive cells (**B**, *P*>0.05). All data are mean ± SD.

To determine whether re-coupling eNOS with simvastatin prevents neuronal death, we assessed brains of mice with SAH treated with vehicle or simvastatin by TUNEL, caspase-3/NeuN staining. Numerous TUNEL-positive neurons were observed in the brains of vehicle-treated SAH mice (Fig. 5). Double-labelling for caspase-3 and NeuN demonstrated a similar pattern of localization and abundance of double positively stained neurons in vehicle-treated animals as seen with TUNEL staining (Fig. 6). The number of TUNEL positive neurons was significantly reduced by treatment with simvastatin started before or after induction of SAH (Fig. 5 and Fig. 7, *P*<0.01 except post-treatment in cortex). Similarly, both pre- and post-SAH simvastatin treatment significantly reduced caspase-3/NeuN double positive neurons in cerebral cortex and hippocampus in comparison to vehicle treatment (Fig. 6 and Fig. 7, *P*<0.05∼0.01).

## Discussion

SAH is associated with oxidative stress due mainly to clot-derived reactive oxygen species [14]. Secondary complications of SAH include large-artery vasospasm, microthromboembolism and neuronal injury, for which multiple mechanisms have been suggested [1], [2]. We previously observed that experimental SAH was associated with increased phosphorylation of Ser1177 in eNOS, but that this was paradoxically accompanied by reduced NO [6]. This was suggestive of eNOS uncoupling. Additionally, SAH was associated with nitrosative stress as shown by increased O_2_Γ and nitrotryosine deposits. We speculated that increased nitrosative stress along with eNOS uncoupling further exacerbates oxidative stress and could be important in contributing to vasospasm, microthromboembolism and neuronal injury [6]. In the present study we provide direct evidence for eNOS uncoupling by demonstrating altered eNOS dimer/monomer ratios in vehicle-treated mice with SAH in comparison to naïve controls. SAH increased phosphorylated, activated eNOS, decreased eNOS dimer and increased eNOS monomer expression. The decreased eNOS dimer/monomer ratio provides direct evidence of eNOS uncoupling after SAH. Both pre- and post-SAH treatment with simvastatin reduced phosphorylation of Ser1177 eNOS and eNOS monomer, suggesting restoration or recoupling of eNOS function. Pre-treatment with simvastatin also abrogated the SAH-induced increase in iNOS. This recoupling of eNOS is associated with alleviation of vasospasm, neuronal apoptosis, microthromboembolism, oxidative stress and restoration of NO/O_2_Γ balance. The results suggest that eNOS activation and its uncoupling may be a key factor in the pathogenesis of vasospasm and secondary complications of SAH such as microthromboembolism [15]. Similar mechanisms have been observed in vascular pathologies like diabetes, hypertension and thrombosis [4]. In these conditions, oxidative stress may be a cause of eNOS uncoupling that is due mainly to activation of nicotinamide adenine dinucleotide phosphate oxidases.

It is noticeable that when compared to the naïve controls, the results seem a little difference between pre- and post-treatment with simvastatin. The difference observed between the ratios from pre- or post-SAH simvastatin groups could be that mice in pre-SAH simvastatin treatment group were better equipped to deal with oxidative stress and had a reduced expression of eNOS monomers, thus a higher ratio in these animals. The modality of treatment was longer thus it is predicted that these animals are likely to have maybe a more robust endothelium and be less likely to suffer endothelial damage. In comparison, the post-treated group has a slightly reduced dimer/monomer ratio, which is caused by the relatively higher expression of eNOS monomers. Despite the difference, post-treated animals had a significantly higher ratio than that in placebo treated animals, indicating that post-SAH treatment is still effective at preventing eNOS uncoupling. Another major difference between the groups is that oxidative stress remained moderately high in the post-SAH simvastatin treated group as demonstrated from the sustained expression of iNOS (Fig. 3E) and superoxide anion radical (Fig. 3C). This could be due to the fact that statin therapy for only 48 hours was not sufficient enough to reduce oxidative stress effectively or fast enough to prevent most eNOS uncoupling, thus making the dimer/monomer ratio level smaller due to the prevalence of monomers. In the pre-SAH treatment group, a longer treatment with simvastatin might confer protective effects on the endothelium as previously documented, thus making its less prone to oxidative damage and uncoupling. The prevention of eNOS uncoupling would also reduce oxidative response and incidence of thrombosis and cell death due to the increased availability of NO and decreased reactive oxygen species.

Prior studies pretreated mice or rats with simvastatin and then induced SAH using the endovascular perforation model [16]–[19]. Simvastatin was shown to alleviate vasospasm [16]–[19], reduce cognitive dysfunction [20] and decrease inflammatory cell infiltration [17]. The decrease in vasospasm is consistent with the present results but prior studies found that treatment with simvastatin beginning before SAH increased eNOS [16]. However, post-SAH treatment with simvastatin decreased vasospasm and improved neurobehavioral deficits but had no effect on eNOS protein level. In contrast, Sugawara, et al., used the same post-SAH simvastatin treatment scheme and the endovascular perforation model in rats and reported that simvastatin not only improved neurological deficits, but also increased the phosphorylated form of eNOS without increasing total eNOS expression [19]. We report that SAH increased eNOS dimer, monomer and phosphorylated forms. However, the increased eNOS was accompanied by decreased NO availability, suggesting eNOS uncoupling. Simvastatin, instead of increasing eNOS expression, decreased the expression of eNOS to the naïve control level, and increased NO production suggesting re-coupling of eNOS. Prior studies did not examine eNOS monomer and dimer or NO concentrations [16], [19]. The discrepancy may be due to the difference between species or the models since the endovascular perforation model causes SAH associated with transient global ischemia that may be greater than that occurring in the prechiamatic injection model. In agreement with our suggested hypothesis and mode of pathogenesis, other reports confirmed some of our findings. Berra et al., found there was a significant increase in iNOS and eNOS in patients with SAH compared to controls, and that this increase was associated with poor clinical condition [21]. Osuka et al., reported that SAH induced by cisternal blood injection in rats was associated with an increase in phosphorylated eNOS one and two days after SAH [22].

The current experiments suggest that SAH itself increases eNOS rather than that vasospasm increases endothelial shear stress which secondarily increases eNOS [14]. The mechanism of increase is uncertain but may involve oxidative stress due to perivascular blood. The increase in phosphorylated eNOS consisted of both dimer and monomer although there was a much greater increase in monomer than in dimer in vehicle-treated SAH animals compared to naïve controls. Thus it is likely that whatever eNOS is produced was constantly uncoupled. Simvastatin decreased both eNOS dimer and monomer to or close to levels seen in naïve mice indicating that simvastatin modulates eNOS levels rather than simply up-regulating the protein as previously reported [16], [19]. During uncoupling, eNOS produces O_2_Γ which reacts with endogenous NO producing ONOO^−^. This depletes NO resulting in microthromboembolism and vasospasm, which in turn contribute to neuronal death.

Regarding the dose of simvastatin used in current study, we adopted from McGirt et al [16], who utilized simvastatin to up-regulate eNOS. Even though we did not examine the dose responses, the dose used here is considered a high dose. However, it falls within the clinical acceptable dose range that can be administered to humans [16]. In experimental settings, Wang et al., showed that high dose of simvastatin (10 and 30 mg/kg) upregulates dopamine D1 and D2 receptors in the cerebral cortex possibly through upregulation of eNOS in Sprague–Dawley rats [23]. Similarly, using the same treatment scheme as in current study (20 mg/kg for 14 days), Entres et al showed that simvastatin has neuroprotective effects against cerebral ischemia in 129/SV mice though upregulation of eNOS [24]. However, similar to abovementioned studies, whether the upregulated eNOS is monomer or dimer or a mixture of both is not known.

The molecular mechanisms underlying the beneficial effect of simvastatin on eNOS are not known. Many potential mechanisms warrant further investigation. It is known that BH4, a potent naturally occurring reducing agent, plays a key role in dimmerization of eNOS [4] [25]. Oxidative stress, which occurs after SAH, may lead to excessive oxidation and depletion of BH4, which in turn could result in uncoupling of eNOS. Other mechanisms such as l-arginine deficiency, increased endogenous eNOS inhibitor asymmetric dimethyl-L-arginine [26]–[27] or disruption of the zinc-thiolate cluster [28] may all contribute to uncoupling of the enzyme. The limitations of these experiments are that the mechanism of eNOS activation after SAH remains to be characterized, although candidate processes include vasospasm [14] and increased oxidative stress [4], both of which occur after SAH [12]. We observed elevated iNOS levels in the brain parenchyma, which may contribute to oxidative stress, ONOO^−^ formation, microthromboembolism and neuronal damage [29]. However, even when iNOS remained elevated in animals treated with simvastatin after SAH, most of secondary complications were decreased, suggesting iNOS may not play a major role in the pathogenesis of vasospasm and other secondary complications [21]. We also refer to microthromboembolism to describe fibrin deposits in microvessels because it is not known if these form in situ or embolize after being formed on the injured endothelium of larger proximal arteries. Finally, statins have multiple biological effects. They stabilize atherothrombotic plaques [30]–[32], reduce inflammation and oxidative stress [33], [34], reduce microvasculature thrombosis and improve endothelial function [35], [36]. These effects are reported to be mediated by multiple mechanisms such as direct scavenging of reactive oxygen species [37], inhibition of expression of matrix metalloproteinases 2 (MMP-2) and MMP-9 [38], [39] inhibition of protein isoprenylation, farnesylation and geranylgeranylation [40]. The pleiotrophic effects of statins mean that we cannot exclude other mechanisms by which they may have alleviated secondary complications of SAH.

Clinical use of statins for the management of SAH is controversial. Retrospective studies reported mixed results, with three studies finding an increased risk of vasospasm or minimal effect in statin-treated patients [11], [13], [41], [42] and three primary studies reporting a marked reduction in vasospasm[43] [17], [44] and a metaanalysis confirming the observed trend of vasospasm reduction[45]. Additionally, two reported improved short-term clinical outcome and decreased mortality, respectively [44], [46]. Similar conflicting results were observed in randomized clinical trials and metaanalyses [47]. It is likely that the variation in clinical benefit is due to the small number of patients treated, and in type of statin used, dose and timing of administration.

In conclusion, our results suggest simvastatin can re-couple dysfunctional eNOS and restore NO/O_2_Γ balance, thus preventing secondary complications, such as vasospasm, microthromboembolism and neuronal injury after SAH in mice. The results also suggest that both pre- and acute post-treatment regimens may be effective at alleviating eNOS uncoupling-associated complications. Furthermore, simvastatin restored eNOS levels to that of naïve mice in terms of both dimer and monomer concentrations, possibly indicating the importance of eNOS in the pathogenesis of complications of SAH.
